# Ophthalmia in the Army.—A Contrast

**Published:** 1897-02-13

**Authors:** 


### OPHTHALMIA IN THE ARMY.—A CONTRAST.

Dr. J. Tweedy, Harley Street, writes: In drawing a contrast at the Mansion House between the effects of ophthalmia in the Egyptian campaigns of about a century ago and that of the year of 1882, I did not wish it to appear that I could personally claim any credit for the impressive and gratifying results. The credit, I believe, belongs almost entirely to Surgeon-General Marston, who by his observations, studies, researches, and reports has probably done more than any other one person to lessen the prevalence of ophthalmia in the British Army. That he should have consulted me at all was only one example, among many, of his wise foresight in endeavouring to prevent, and, failing that, to mitigate the effects of an appalling contingency.

